# Specimen oriented intraoperative margin assessment in oral cavity and oropharyngeal squamous cell carcinoma

**DOI:** 10.1186/s40463-021-00501-5

**Published:** 2021-06-21

**Authors:** P. Horwich, C. MacKay, M. Bullock, S. M. Taylor, R. Hart, J. Trites, L. Geldenhuys, B. Williams, M. H. Rigby

**Affiliations:** 1 Department of Surgery, Division of Otolaryngology – Head and Neck Surgery, Queen Elizabeth II Health Science Centre and Dalhousie University, 3rd Floor Dickson Building, VG Site, 5820 University Avenue, Halifax, Nova Scotia B3H 2Y9 Canada; 2 Department of Pathology, Division of Anatomical Pathology, Queen Elizabeth II Health Science Centre and Dalhousie University, Halifax, Nova Scotia Canada

**Keywords:** Frozen section, Margin status, Oral cavity, Oropharynx, Squamous cell carcinoma

## Abstract

**Objective:**

Evaluate the oncologic outcomes and cost analysis of transitioning to a specimen oriented intraoperative margin assessment protocol from a tumour bed sampling protocol in oral cavity (OCSCC) and oropharyngeal squamous cell carcinoma (OPSCC).

**Study design:**

Retrospective case series and subsequent prospective cohort study

**Setting:**

Tertiary care academic teaching hospital

**Subjects and methods:**

Retrospective case series of all institutional T1-T2 OCSCC or OPSCC treated with primary surgery between January 1st 2009 – December 31st 2014. Kaplan-Meier survival estimates with log rank tests were used to compare patients based on final margin status. Cost analysis was performed for escalation of therapy due to positive final margins. Following introduction of a specimen derived margin protocol, successive prospective cohort study of T1-T4 OCSCC or OPSCC treated with primary surgery from January 1st 2017 – December 31st 2018. Analysis and comparison of both protocols included review of intraoperative margins, final pathology and treatment cost.

**Results:**

Analysis of our intra-operative tumour bed frozen section protocol revealed 15 of 116 (12.9%) patients had positive final pathology margins, resulting in post-operative escalation of therapy for 14/15 patients in the form of re-resection (7/14), radiation therapy (6/14) and chemoradiotherapy (1/14). One other patient with positive final margins received escalated therapy for additional negative prognostic factors. Recurrence free survival at 3 years was 88.4 and 50.7% for negative and positive final margins respectively (*p* = 0.048). Implementation of a specimen oriented frozen section protocol resulted in 1 of 111 patients (0.9%) having positive final pathology margins, a statistically significant decrease (*p* < 0.001). Utilizing our specimen oriented protocol, there was an absolute risk reduction for having a final positive margin of 12.0% and relative risk reduction of 93.0%. Estimated cost avoidance applying the specimen oriented protocol to our previous cohort was $412,052.812017 CAD.

**Conclusion:**

Implementation of a specimen oriented intraoperative margin protocol provides a statistically significant decrease in final positive margins. This change in protocol leads to decreased patient morbidity by avoiding therapy escalation attributable only to positive margins, and avoids the economic costs of these treatments.

**Graphical abstract:**

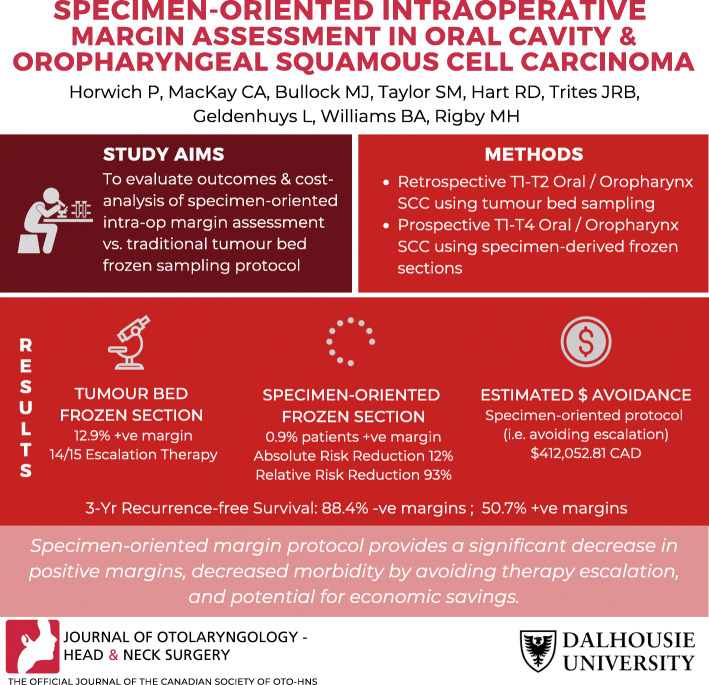

**Supplementary Information:**

The online version contains supplementary material available at 10.1186/s40463-021-00501-5.

## Background

Failure to eradicate disease at the primary site has been reported as the single largest cause of mortality in head and neck cancer [1]. This is particularly true for early stage squamous cell carcinoma (SCC) of the oral cavity and oropharynx, which ideally can be treated with surgical resection as a single modality provided favourable pathology and negative margins. Negative frozen sections intra-operatively with positive final pathology can lead to significant patient morbidity and potentially mortality. These false negative frozen sections expose patients to further treatment modalities including re-resection, chemotherapy and/or radiation which results in additional treatment costs and financial burden on the healthcare system.

The impact of a positive margins on final pathology in head and neck cancer ablation cannot be overstated. Multiple studies have reported the strongest predictor of local recurrence, disease specific survival and overall survival was the final tumour margin status [1–11]. A recent large review published by Smits et al. demonstrated statistically significant negative impacts of positive margins in oral cavity SCC on local recurrence, regional recurrence, distant metastasis and overall survival [9]. Binahmed et al. reported a cohort of 425 patients with oral cavity SCC, demonstrating that a positive margin status resulted in a 90% increased risk of death at 5 years [4]. These outcomes occur despite the fact that positive margins on final pathology result in an escalation of care for patients. This includes potential re-resection, radiotherapy, chemotherapy or a combination of the three. This escalation of care is costly, and places a substantial financial burden on the healthcare system [4, 6, 12, 13].

Even for patients who are salvaged with treatment escalation (i.e. repeat surgery, adding or increasing the dose of radiation, or the addition of chemotherapy) the increased intensity of treatment comes with significant additional morbidity [4, 6, 14, 15]. Binahmed et al. observed minor (33.1%) and major morbidity (7.9%) in 127 patients who received adjuvant radiation therapy delivered by conventional fractionation [4]. The morbidity levels for patients who received radiation therapy were significantly higher than the minor and major morbidity levels (7.7 and 2.7% respectively *p* < 0.0001) in those who were treated with surgery alone. Similarly, Nason et al. recorded significantly more complications in patients who received adjuvant radiation therapy (43%) compared to those who received surgery only (13%, *p* < 0.001) [6]. With regard to dose, Peters et al. found that moderate to severe complications associated with postoperative radiation therapy occurred more frequently in patients who received ≥63 Gy [14]. In a meta-analysis of adjuvant chemotherapy trials El-Sayed and Nelson found that the addition of chemotherapy significantly increased toxicity (*p* < 0.001) [15]. Specifically, nausea, mucositis, delayed radiotherapy, treatment-related death, bone marrow toxicity, and overall toxicity were all significantly more likely to occur with the addition of adjuvant chemotherapy.

Frozen section analysis has been found to be an accurate, time sensitive method of intra-operatively evaluating oncologic resection margins [16–18]. Significant debate in the literature remains on where best to send frozen sections from, with the remaining tumour bed or resected specimen oriented samples being the most common. In a 2005 survey of American Head and Neck Society members, 76% of surgeons exclusively sent defect driven frozen sections from the tumour bed [17].

Final mucosal margins are rarely positive, and one study demonstrated isolated positive mucosal margins in less than 2% of cases [19]. These margins are also much more amenable to intraoperative analysis via frozen section, and a representative circumferential sample from the main resection can usually be achieved. In contrast, the deep margin is much more frequently positive, and is much more difficult to sample in a representative way [19].

There is increasing evidence that a specimen oriented analysis reliably leads to more accurate determination of margins and is associated with improvements in local control and overall survival [18, 20–22]. In similar studies of patients with pT1–2 pN0 oral tongue cancer, Chang et al. and Maxwell et al. both found that there was a greater probability of local progression-free or local recurrence-free survival at 3 years when specimen oriented sampling was used compared to defect-driven sampling (0.9 vs. 0.73, *p* = 0.0389 and 0.9 vs. 0.8, *p* = 0.03 respectively) [18, 20]. In addition, Chang demonstrated no correlation between defect driven frozen section results and final margin status [20]. Amit et al. found that using specimen oriented sampling for intraoperative frozen sectioning, the rate of final negative surgical margins was 84% compared to 55% (*p* = 0.02) using defect-driven sampling [22]. Until January 2017, specimen oriented analysis had not been routinely done at the QEII health science centre for head and neck cancer.

**Fig. 1:**
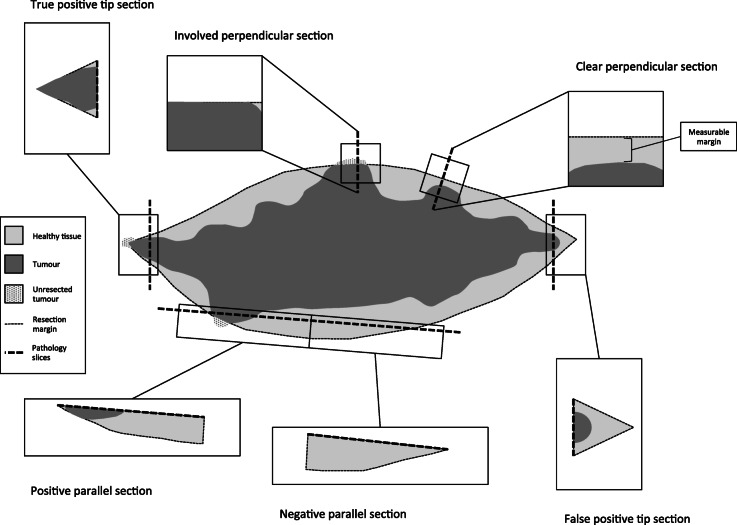
Margin orientation and analysis

An additional benefit of specimen oriented margins is the ability to measure the distance from the tumour to the margin of interest. In contrast, defect-oriented margins can only be reported as positive or negative [8]. To measure distance, pathological sectioning should be done perpendicular to the margins of interest so that the distance from the tumour to the surgical margin can be measured (see Fig. 1). Sections that are done parallel to the margin give a larger surface area but does not allow for the measurement of margin distance [8] (see Fig. 1). Tip margins taken from a specimen are technically easier to harvest, but do not give distance to the margin, and can give false positives of tumour involvement of the true surgical margin (see Fig. 1).

Varvares et al. recently provided further evidence that margins ≥5 mm are associated with better outcomes in oral cancer [21]. Patients that had margins ≥5 mm had a recurrence rate of only 3.4% compared to patients with margins < 5 mm or with positive margins resected to negative which were 26.4 and 28.6% respectively. Specimen driven analysis at time of surgery allows potential revision of margins less than 5 mm as well as positive margins.

Re-resection of positive margins to negative margins has been demonstrated to be effective in decreasing the associated morbidity and mortality. Studies by both Karatzanis et al. and Jäckel et al. reviewing outcomes for glottic cancer and other head and neck subsites have shown that for patients that had initially positive margins re-resected to negative margins did equally well as patients who had negative margins from the outset [23, 24]. In contrast, one study using tumour bed driven frozen section was unable to show benefit of re-resection of a positive frozen section intra-operatively [10]. The authors propose that a positive margin, even when re-resected to negative status may be acting as a surrogate marker for more aggressive disease impacting recurrence and survival [10]. This is potentially supported by the findings of Patel reported in 2010, demonstrating that for oral cavity cancers that were N0, tumours requiring re-resection to achieve negative margins had similar 5-year local control, regional control, and disease specific survival as tumours initially resected with clear margins. In contrast, for more aggressive tumours with N1–2 disease there was an associated decreased 5-year local control, regional control and disease specific survival in cases where re-resection was required to achieve negative margins when compared to cases where clear margins were achieved on initial resection [25].

The purpose of this study is to review and determine the efficacy of our prior institutional tumour bed driven intraoperative margin assessment, and the impact of implementing a specimen oriented protocol. Oncologic outcomes and treatment costs associated with final positive margins in T1-T2 oral cavity (OCSCC) and oropharyngeal squamous cell carcinoma (OPSCC) are also evaluated.

## Methods

An initial retrospective case series of all patients with T1-T2 OCSCC or OPSCC treated with primary surgery at the QEII Health Sciences Center in Halifax Nova Scotia was performed. A data request through the centralized provincial cancer database, Cancer Care Nova Scotia, was performed identifying all patients treated between January 1st 2009 – December 31st 2014. Patients treated with primary chemoradiation therapy, primary radiation therapy and patients with recurrence (defined as previous treatment for SCC within 5 years) were excluded. Patients treated surgically by services other than Otolaryngology – Head and Neck surgery were also excluded due to the protocol changes being department specific. Oropharyngeal resections were completed using either transoral laser microsurgery (TLM) using the CO2 laser, or transorally using monopolar cautery based on surgeon preference. A database was created and individual charts were reviewed collecting baseline patient characteristics, tumour stage and subsite and follow up data. For the defect driven cohort, all intra-operative frozen sections sent were defect driven from the residual tumour bed, as per our institutional standard at the time. Both circumferential margins and deep margins were evaluated in this manner. In the setting of a positive intra-operative frozen section, re-resections were completed until the frozen section was negative. Pathology reports were reviewed for intra-operative frozen section margin results and final pathology margin status. Kaplan-Meier 36-month survival estimates with log rank tests were used to compare patients based on final margin status for the following endpoints: overall survival (OS) defined as death to due to any cause, local control (LC) defined as time to local recurrence, and recurrence free survival (RFS) defined as time to any recurrent disease. The decision to escalate care in the form of re-resection, radiation therapy or chemoradiotherapy is made by our institutional head and neck cancer multidisciplinary tumour board on a case by case basis, following published NCCN guidelines [26].

Cost analysis was performed for escalation of therapy attributable only to positive final margins. Adjuvant therapies that would have been done for other tumour characteristics (ex. chemoradiation for extranodal extension) were not included as attributable to a positive margin. Treatment cost was quantified taking into account multiple institutional specific variables for intention to treat via re-resection, radiotherapy and chemoradiotherapy. Variables included in cost analysis were operating room time and supplies, nursing costs, surgeon and anesthesiologist fees, anesthesia consumables, pathologist fees, radiation treatment time and cost of chemotherapeutics. A detailed itemized cost analysis per escalation of therapy can be found in Additional files 1, 2 and 3.

Specimen oriented frozen section analysis protocol began on January 1st, 2017 and prospective results were collected using database variables from the retrospective defect oriented study. The protocol itself involves resecting the specimen en bloc or via a tumour split by the staff surgeon. The specimen(s) are taken to the pathology lab and immediately examined in the presence of the surgeon and the pathologist. A standardized resection template is used to help orient the specimen and the surgeon indicates areas of most concern to avoid miscommunication with the pathologist (Additional file 4). Any tumour splits are identified and inked. The remaining specimen margins are then inked by the surgeon and pathologist together. The specimen is then grossed by cutting perpendicular to the tumour/margin interface to maximize the ability to analyze the distance and relationship of the tumour with the deep margin. If gross disease is noted at, or close (< 5 mm) to a deep margin, re-resection will be performed prior to frozen section analysis. In the absence of a close or positive margin, frozen section margins from the specimen itself are harvested and assessed. During this analysis pathologists assess both margin positivity and, if negative, distance of the tumour to the deep margin. In the setting of a positive frozen section, re-resections were completed until the frozen section was negative. For close margins, re-resections were performed on a case by case basis, depending on surgeon judgment of the morbidity and feasibility of re-resection. For our prospective analysis, our inclusion criterion was expanded to include T1 – T4 OCSCC or OPSCC, treated with primary surgery between January 1st, 2017 and December 31st, 2018. Patients with bony invasion were excluded from our analysis. All patients treated with primary chemoradiation or radiation therapy, and patients with recurrence defined as previous treatment for SCC within 5 years, were excluded. Analysis included intra-operative and final pathology reports, and results were compared between protocols. Fisher’s exact test was used to determine if there was a statistically significant difference between the final positive margin rates of the two protocols.

Ethics approval for the study was obtained from the Nova Scotia Health Authority Research Ethics Board (ROMEO #1020700).

## Results

### Retrospective study results

**Fig. 2:**
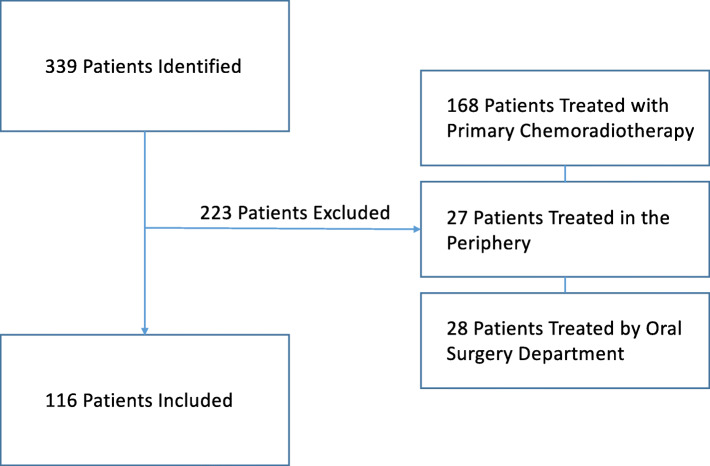
Retrospective cohort of provincial T1–2 OCSCC and OPSCC treated from January 1st 2009 – December 31st 2014. Inclusion and exclusion factors

There were 339 patients identified from the provincial cancer database prior to January 2017. All charts were individually reviewed by PH with 116 patients meeting inclusion criteria (Fig. 2). The majority of patients were male gender (69%) with a past smoking history (97%). Seventy-three patients had primary OCSCC (63%) while 43 had primary OPSCC (37%). Baseline patient characteristics for the retrospective analysis are illustrated in Table 1.

**Table 1:** Retrospective analysis baseline patient characteristics

Patient Characteristic	Value (***n*** = 116)
Male	80 (69%)
Female	36 (31%)
Mean Age	63.2 (Range 38–88)
History of Smoking	113 (97%)
Oral Cavity	73 (63%)
T1	29 (25%)
T2	44 (38%)
Oropharynx	43 (37%)
T1	19 (16%)
T2	24 (21%)

Fifteen patients (12.9%) were identified that had either no margins taken, or negative frozen section margins but positive final pathological margins using our original tumour bed sampling protocol. Three of the 15 patients did not have frozen sections sent intra-operatively – all were OCSCC, two were found to be T2 while one was T1. Of these 15 patients with positive final margins, the average number of frozen sections sent was 4.1 (range 0–7), with the majority being T2 lesions (80%). All 15 patients had a positive deep margin. Specific pathological locations are reported in Table 2.

**Table 2:** Positive final pathology margin characteristics

Characteristics	Patients
T1 Lesions	3/15 (20%)
T2 Lesions	12/15 (80%)
Average Number of Frozen Sections	4.1 (Range 0–7)
**Primary Oral Cavity**	9/15 (60%)
Deep Lateral Tongue	3/9 (33%)
Deep Floor of Mouth	4/9 (44%)
Periosteal Alveolar Ridge	1/9 (11%)
Deep Tongue Base	1/9 (11%)
**Primary Oropharynx**	6/15 (40%)
Deep Pharynx	3/6 (50%)
Deep Tongue Base	3/6 (50%)

### Retrospective study survival and oncologic outcomes

**Fig. 3:**
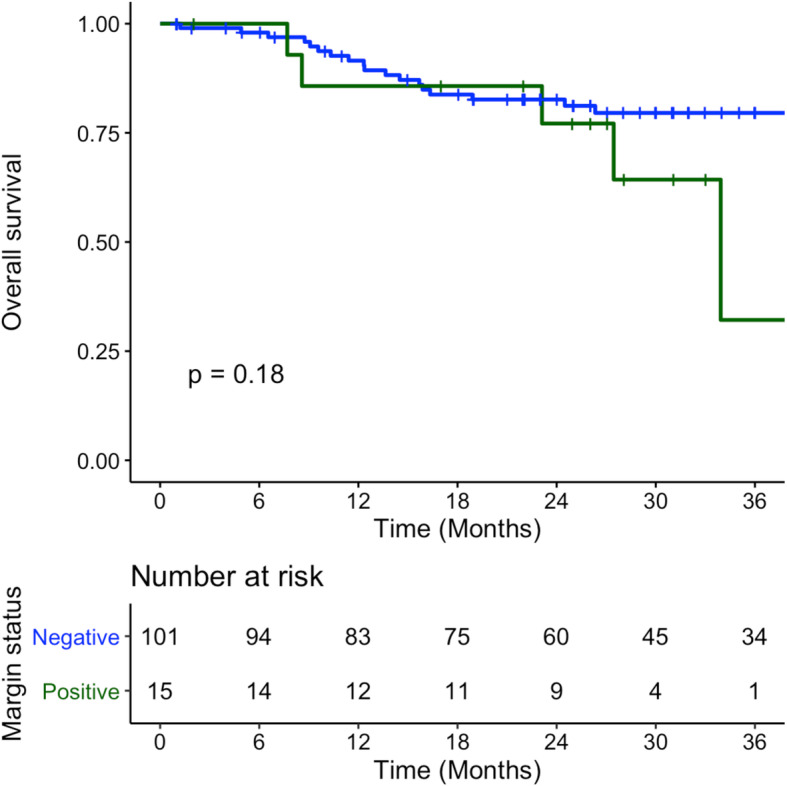
Overall Survival of patients with T1-T2 OCSCC and OPSCC treated with surgery between January 2009 – December 2014 – Blue denotes negative final pathology margins, Green denotes positive final pathology margins

Overall survival at 3 years was 79.6% (SE 4.4%) and 32.1% (SE 24.0%) for negative and positive margins respectively (*p* = 0.175) (Fig. 3). Kaplan-Meier mean overall survival time was 60.3 months (SE 4.1) for negative margins and 30.5 months (SE 3.2) for positive margins.

**Fig. 4:**
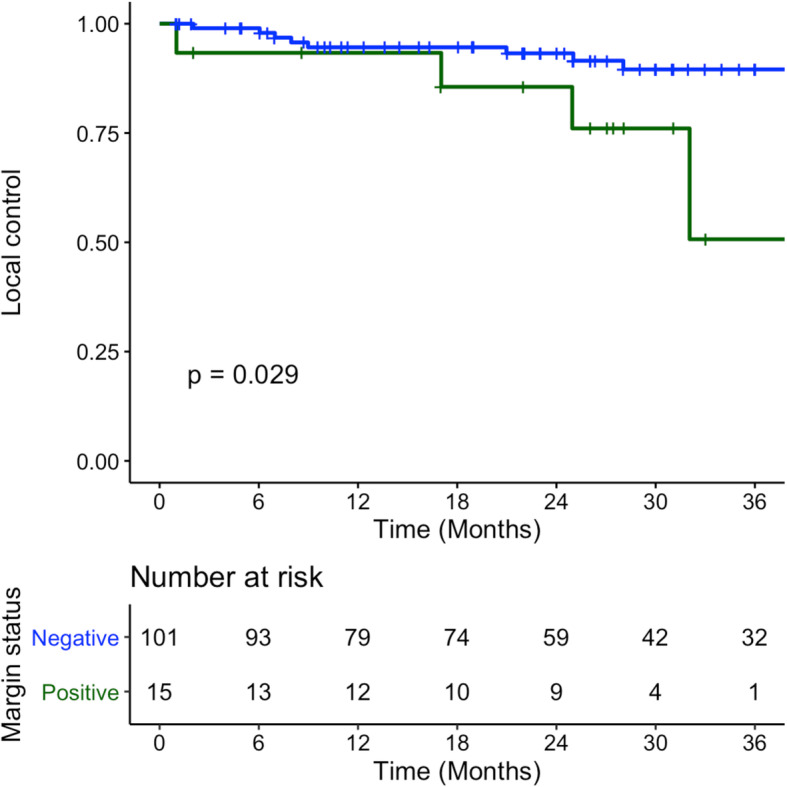
Local Recurrence of patients with T1-T2 OCSCC and OPSCC treated with surgery between January 2009 – December 2014 – Blue denotes negative final pathology margins, Green denotes positive final pathology margins

Local control at 3 years was 89.5% (SE 3.6%) and 50.7% (SE 22.3%) for negative and positive margins respectively (*p* = 0.029) (Fig. 4). Kaplan-Meier mean time of local control was 73.8 months (SE 3.6) for negative margins and 32.2 months (SE 3.3) for positive margins.

**Fig. 5:**
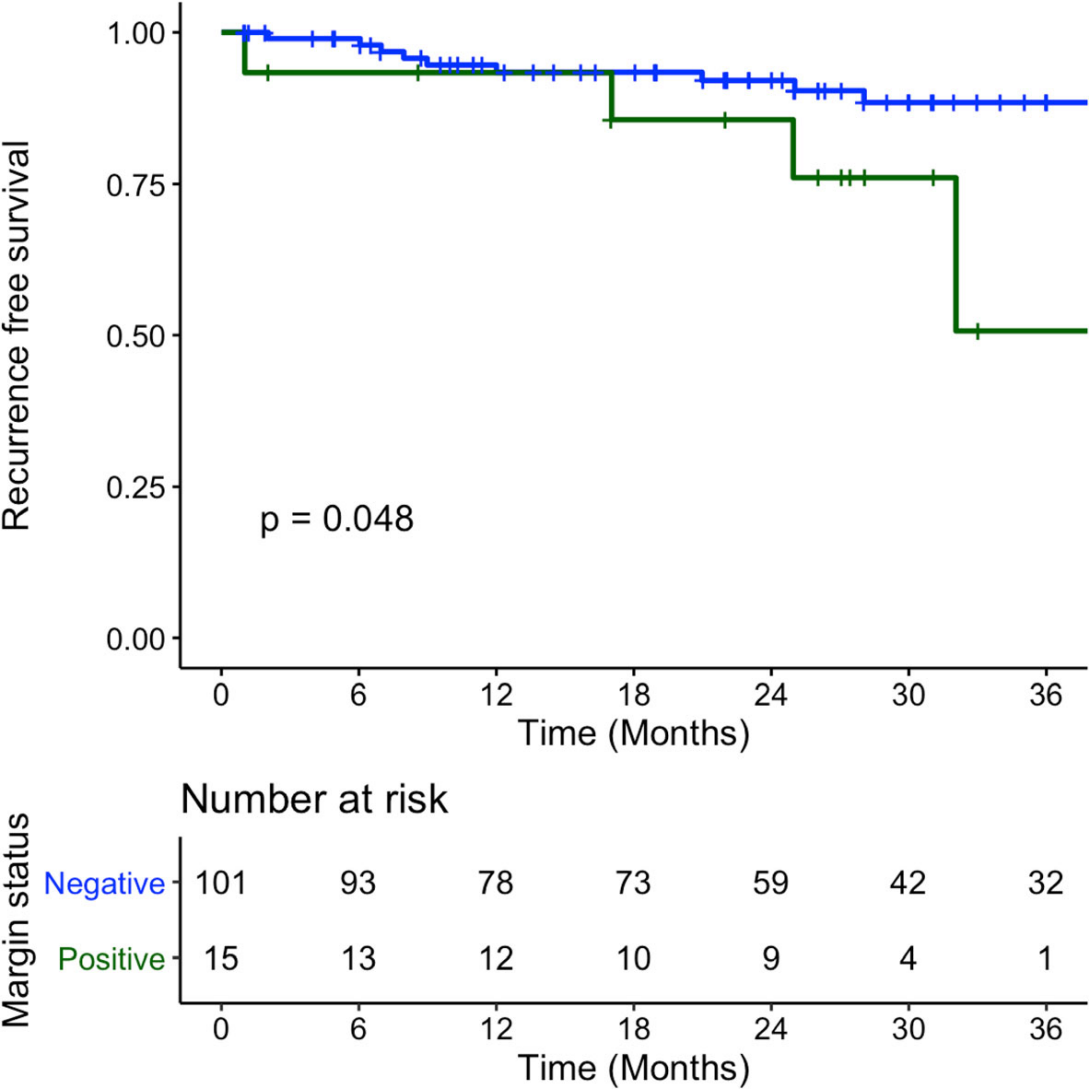
Recurrence Free Survival of patients with T1-T2 OCSCC and OPSCC treated with surgery between January 2009 – December 2014 – Blue denotes negative final pathology margins, Green denotes positive final pathology margins

Recurrence free survival at 3 years was 88.4% (SE 3.8%) and 50.7% (SE 22.3%) for negative and positive margins respectively (*p* = 0.048) (Fig. 5). Kaplan-Meier mean time of recurrence free survival was 73.0 months (SE 3.6) for negative margins and 32.2 months (SE 3.3) for positive margins.

Fourteen of 15 patients with positive final pathological margins had escalation of therapy primarily attributable to positive margin status. Pathological characteristics for each patient with a positive final margin can be found in Table 3. Seven had re-resection, 6 had radiation therapy and 2 had chemotherapy.

**Table 3:** Pathological characteristics of final pathology and escalation of therapy (*n* = 15). Patient marked with asterisk (*) excluded from cost analysis

Patient Number	Margin Status	Perineural Invasion (PNI)	Lymphovascular Invasion (LVI)	Extracapsular Extension (ECE)	Adjuvant Therapy
1	+	–	–	–	Radiation
2	+	+	–	–	Re-resection
3	+	–	–	–	Re-resection
4	+	–	+	–	Radiation
5	+	+	–	–	Re-Resection
6	+	+	+	–	Chemoradiotherapy
7	+	–	–	–	Radiation
8	+	–	–	–	Re-Resection
9	+	–	–	–	Radiation
10*	+	+	+	+	Chemoradiotherapy
11	+	–	–	–	Re-Resection
12	+	–	–	–	Re-Resection
13	+	–	–	–	Radiation
14	+	–	–	–	Re-Resection
15	+	–	–	–	Radiation

### Retrospective study cost analysis

Mean cost per re-resection of a positive margin was $20,688.582017 CAD, with a mean operating time of 215 min per case. Mean cost for radiation therapy of a positive margin (66 Gray in 33 fractions) was $40,962.672017 CAD. Mean cost of chemoradiotherapy (concurrent cisplatin + 66 Gray in 33 fractions) treatment due to a positive margin was $52,360.70 CAD. The cumulative cost estimate of escalation of therapy due to positive margins on final pathology for our 14 patient subset was $442,956.772017 CAD (Table 4).

**Table 4:** Adjuvant treatment and cost attributable to positive margin

Adjuvant Treatment Type	Number of Patients (***n*** = 14)	Mean Cost per Treatment (2017 CAD)	Cumulative Cost (2017 CAD)
Re-Resection	7	$20,688.58	$144,820.07
Radiation Therapy	6	$40,962.67	$245,776.00
Chemoradiotherapy	1	$52,360.70	$52,360.70
			Total - $442,956.77

### Prospective study results

Our prospective analysis identified 130 patients between January 1st 2017 and December 31st 2018. One hundred eleven patients met inclusion criteria, with 19 patients excluded for unknown primary not being identified (*n* = 10), the new protocol not being used (*n* = 6), and the primary site being not oral cavity or oropharynx on final review (*n* = 3).

The majority of our prospectively collected patients were similarly male (72%). Forty-six patients had primary OCSCC (41%) while 65 had primary OPSCC (59%). Baseline characteristics for our prospective data can be found in Table 5. Nodal status for our prospective and retrospective cohorts can be found in Table 6.

**Table 5:** Prospective analysis patient baseline characteristics

Patient Characteristic	Patients (***n*** = 111)
Male	80 (72%)
Female	31 (28%)
Mean Age	61.7 (Range 37–83)
Oral Cavity	46 (41%)
T1	16 (14%)
T2	18 (16%)
T3	8 (7%)
T4a	4 (4%)
Oropharynx	65 (59%)
T1	24 (22%)
T2	29 (26%)
T3	9 (8%)
T4	3 (3%)

**Table 6:** Retrospective and prospective nodal staging

Nodal Stage	Retrospective (***n*** = 116)	Prospective (***n*** = 111)
N0	65 (56%)	40 (36%)
N1	12 (10%)	33 (30%)
N2		2 (2%)
a	11 (9%)	6 (5%)
b	22 (19%)	18 (16%)
c	2 (2%)	3 (3%)
N3	3 (3%)	4 (4%)
Unknown	1 (1%)	5 (4%)

Using our specimen oriented protocol, 1/111 (0.9%) of patients had positive final pathology in the setting of negative frozen section margins. This patient had an anterior floor of mouth lesion involving the alveolar ridge. On frozen section the periosteum was not felt to be involved. A rim mandibulectomy was not performed. Deeper sections in the final analysis demonstrated involvement of the periosteum, with tumour at the deep aspect of the specimen in the area of involvement. This patient underwent escalation of therapy in the form of an increased dose of radiotherapy, 66 Gy in 33 fractions.

**Fig. 6:**
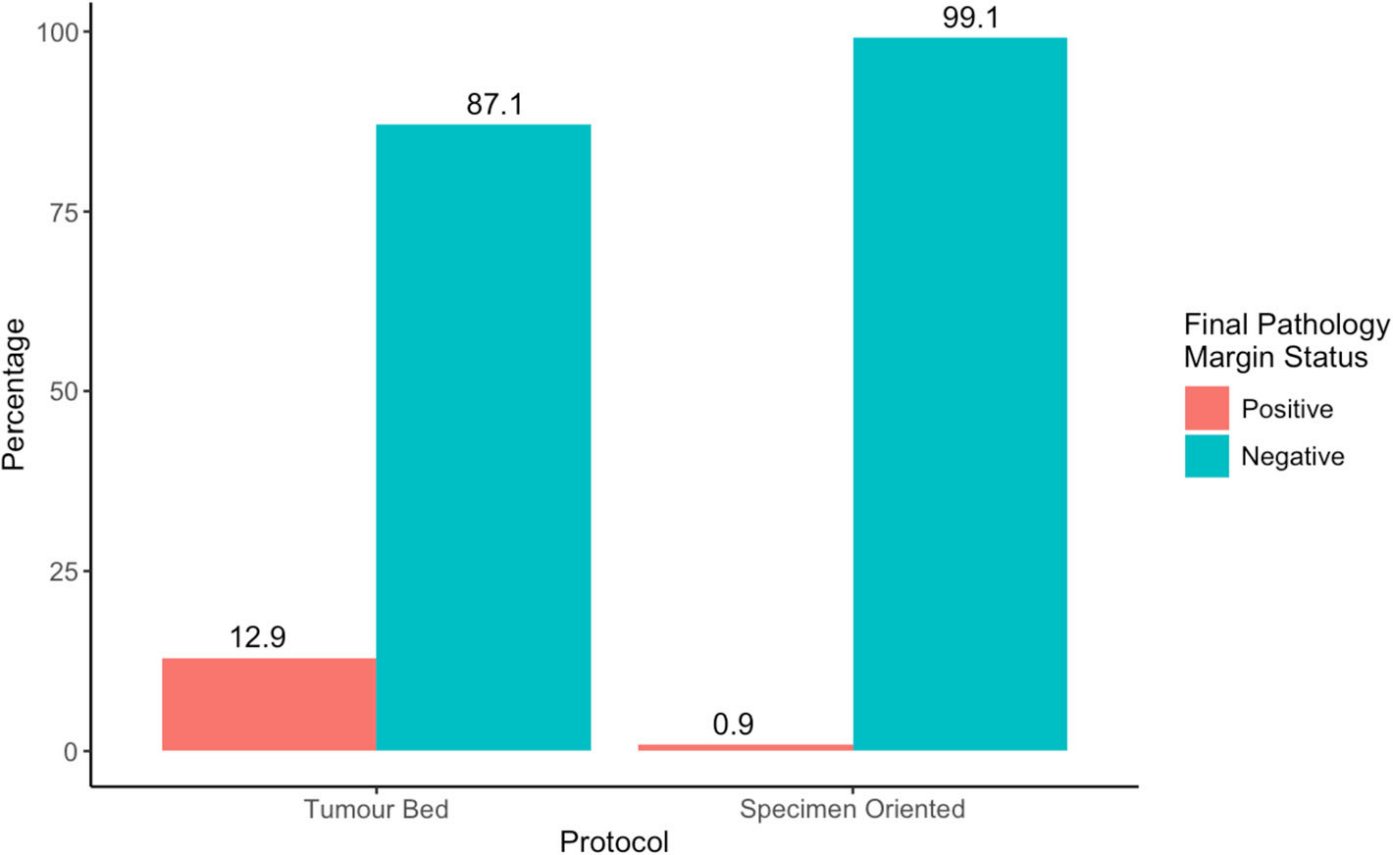
Tumour bed vs specimen oriented protocol comparison of final margin positivity

The decrease in final margin positivity to 0.9% using the specimen oriented protocol from 12.9% using our tumour bed driven protocol was statistically significant (*p* = 0.00035) (Fig. 6). Comparing both protocols, this corresponded to an absolute risk reduction of 12.0% (11.8–12.2, 95% CI) and a relative risk reduction of 93.0% (92.3–93.5, 95% CI) of having negative intra-operative frozen sections with positive final pathology margins. Estimated cost avoidance applying the specimen oriented positive margin rate to our previous cohort was $412,052.812017 CAD.

A case matched subset of 28 patients from each intraoperative method were selected to compare the difference in frozen section time for the frozen section pathologists. Mean processing time for the defect oriented method was 44.9 min (SE 2.6). Mean processing time for the specimen oriented method was 50.4 min (SE 4.6). This does not represent a statistically significant difference in processing time between the two methods (*p* = 0.304).

## Discussion

Frozen section analysis is heavily relied upon intra-operatively to ensure complete oncologic resection in the head and neck. Our study is one of few in the literature to evaluate the morbidity and mortality impact of false negative frozen sections, and the first to our knowledge to quantify the cost due to potentially avoidable escalation of therapy. Our study also represents the first institution in Canada to report on the implementation of an intra-operative specimen oriented margin protocol.

Using a tumour bed sampling model of frozen section, our institution demonstrated a 12.9% positive final pathology rate over a 7-year span for T1 and T2 OCSCC and OPSCC. This is on the low end of the range of literature values for defect driven frozen section in head and neck oncology. Maxwell et al. reported a false negative rate of 24% in defect driven frozen sections for T1-T2 oral tongue SCC [18]. Chambers et al. demonstrated a false negative rate of 19.5% in a 2005 case series published in Laryngoscope for advanced disease [27]. Using similar tumour bed sampling, a 2018 study by Baumeister et al. reported of 235 oncologic resections with negative intra-operative frozen sections, 66% had a final pathology margin of < 1 mm [28]. The oncologic three-dimensional defect of the oral cavity or oropharynx provides significantly increased surface area at the tumour bed site. This makes adequate and representative frozen section sampling from the defect itself challenging to ensure negative margins [19]. Our current study highlights this weakness of tumour bed sampling as all our false negatives frozen sections were found at deep locations (Table 2).

Several groups have recently advocated for a specimen oriented model of intra-operative frozen section analysis reporting decreased false negative rates and improved patient outcomes [8, 10, 18, 20–22, 28]. Intra-operative specimen oriented protocols require more resources than tumour bed protocols [8]. A similar specimen oriented protocol published by Hinni et al. also recommends the staff surgeon personally hand off the specimen to the pathologist for discussion of orientation and participating in the margin inking process for complex specimens [8]. Temporarily displacing a staff surgeon mid-surgery does come at a cost to both the patient and system, however this is certainly less than the cost of a revision surgery or other adjuvant therapy secondary to a positive margin. Another resource concern is the potential that additional time is required for frozen section pathologists to process specimen oriented samples. Once the specimen oriented protocol had been implemented, the mean time difference necessary for the frozen section pathologist to help orient the specimen in real time was 5.5 min, and was not significantly different than our previous tumour bed sampling protocol.

In our defect oriented cohort, statistically significant differences were found for local control (89.5% vs 50.7% *p* = 0.029) and recurrence free survival at 3 years (88.4% vs 50.7% *p* = 0.048) comparing negative and positive margins respectively. This difference was observed despite these patients undergoing escalation of therapy in the form of either re-resection, radiation or chemotherapy. Positive final pathology margins, despite subsequent revision surgery, have been shown in the past to confer worse local outcomes in early SCC of the oral tongue [20]. Much of this effect is likely due to the presence of residual disease. In addition, positive margins occur more frequently in the setting of aggressive tumour biological factors including perineural invasion, lymphovascular invasion or oncologic field changes that also impact local control and recurrence [20]. As illustrated in Table 3, the majority (10/15) of patients had no other high-risk pathological features aside from a positive margin leading to escalation of therapy. Follow-up and data collection is ongoing for our prospective specimen oriented cohort in order to compare survival and recurrence outcomes stratified by HPV status and stage.

Only 1 of 111 patients were found to have positive final pathology with negative frozen section margins using our specimen oriented protocol. This is significantly fewer positive margins than our tumour bed driven protocol, and led to a relative risk reduction of 12.0% and a relative risk reduction of 93.0%. Specimen oriented protocols have been compared to tumour bed protocols in the past, the majority favoring the former [18, 20, 22]. A prospective randomized control trial by Amit et al. demonstrated a statistically significant decrease in final margin positivity using a specimen oriented protocol [22]. Mx et al. also compared the two sampling techniques and reported a local recurrence and long term survival benefit of specimen oriented margins [18].

In a resource limited environment such as the Canadian healthcare system, care must be taken to limit potentially avoidable costs. Intra-operative frozen section margin analysis has been previously reported to cost up to $3123 USD per patient [16]. This study demonstrates a cumulative cost avoidance for escalation of therapy secondary to false negative frozen sections of $412,052.812017 CAD (or approximately $29,432.32 per positive margin) when comparing protocols. This demonstrates a cost benefit of utilizing specimen oriented frozen section analysis intra-operatively and emphasizes the fiscal importance of avoiding false negative samples. By improving intraoperative accuracy of frozen section using a specimen oriented model, significant cost benefits to our healthcare system can be expected.

Two possibilities exist with regards to false negative frozen sections from a tumour bed sampling model. The first being that the frozen sections sent are truly disease free but the sampling technique did not adequately identify the area of margin positivity. This possible error illustrates the poor validity of defect driven sampling, and is supported through recent literature [4, 8, 18, 20, 21]. A second potential error is present in both techniques, and is where the frozen section did have disease present, however the sections read by our pathology team were negative. This is more likely with the specimen driven protocol due to a larger bulk of tissue submitted. This was the case on the sole positive margin in the specimen driven analysis group. This concern is minor compared to increasing the validity of the intraoperative analyses, as evidenced by the much lower rate of final margin positivity observed in the specimen driven analysis group.

Another finding of interest in our study was that there were 16 cases with no intraoperative margin assessment via frozen section in our retrospective analysis. Of these, three (18.7%) had positive margins on final pathology. This highlights that surgeon confidence alone in the absence of frozen sections is often inadequate to achieve negative margins in early OCSCC and OPSCC. Given that the deep margins were positive in these cases, it is unclear if the positive margin would have been identified if defect driven frozen sections were taken during these cases.

## Conclusions

Specimen oriented intraoperative margin assessment provides a statistically significant decrease in final positive margins compared to tumour bed sampling protocols. This change in protocol leads to decreased patient morbidity by avoiding therapy escalation, and avoids the economic costs of these treatments.

## Supplementary Information

**Additional file 1.** Itemized Cost of Re-Resection.

**Additional file 2.** Itemized Cost of Radiation Therapy.

**Additional file 3.** Itemized Cost of Chemoradiotherapy.

**Additional file 4.** Oropharyngeal Specimen Orientation Template.

## Data Availability

All datasets analyzed in this manuscript are available on request.

## Abbreviations

OCSCC: Oral cavity squamous cell carcinoma
OPSCC: Oropharynx squamous cell carcinoma
SCC: Squamous cell carcinoma
NCCN: National Comprehensive Cancer Network
OS: Overall survival
LC: Local control
RFS: Recurrence free survival
SE: Standard error
CAD: Canadian Dollars
ICU: Intensive Care Unit
