# Supplementary material for: Specimen oriented intraoperative margin assessment in oral cavity and oropharyngeal squamous cell carcinoma
Source: J Otolaryngol Head Neck Surg. 2021 Jun 21;50:37. doi: 10.1186/s40463-021-00501-5 (PMC8218466; doi:10.1186/s40463-021-00501-5)
Supplement: Supplementary file 1 — Additional file 1. Itemized Cost of Re-Resection. [file 40463_2021_501_MOESM1_ESM.docx]

| **Variable** | **Mean Cost per Case**  **(CAD)** | **Cumulative Cost n = 7**  **(CAD)** |
| --- | --- | --- |
| Operating Room Time (Supplies + Nursing) | $1,382.45 | $9,677.15 |
| Hospital Admission Cost  (ICU + Floor) | $15,494.40 | $108,460.80 |
| Surgeon Fees | $1,741.96 | $12,193.74 |
| Anesthesia Fees (including consumables) | $894.77 | $6,263.38 |
| Pathologist Fees (Frozen Section + technician) | $1,175.00 | $8,225.00 |
|  |  | Total - $144,820.07 |
