# Supplementary material for: Specimen oriented intraoperative margin assessment in oral cavity and oropharyngeal squamous cell carcinoma
Source: J Otolaryngol Head Neck Surg. 2021 Jun 21;50:37. doi: 10.1186/s40463-021-00501-5 (PMC8218466; doi:10.1186/s40463-021-00501-5)
Supplement: Supplementary file 2 — Additional file 2. Itemized Cost of Radiation Therapy. [file 40463_2021_501_MOESM2_ESM.docx]

| **Variable** | **Mean Cost per Patient**  **(CAD)** | **Cumulative Cost n = 6**  **(CAD)** |
| --- | --- | --- |
| Initial Consult | $865.00 | $5,190.00 |
| CT Simulation Clinic Visit | $865.00 | $5,190.00 |
| Weekly Clinic Visits during Treatment | $4325.00 | $25,950.00 |
| Radiation Treatment 66 Gray in 33 fractions  (Fees + Technician + Facility) | $34,907.67 | $209,446.00 |
|  |  | Total - $245,776.00 |
