# Supplementary material for: Specimen oriented intraoperative margin assessment in oral cavity and oropharyngeal squamous cell carcinoma
Source: J Otolaryngol Head Neck Surg. 2021 Jun 21;50:37. doi: 10.1186/s40463-021-00501-5 (PMC8218466; doi:10.1186/s40463-021-00501-5)
Supplement: Supplementary file 3 — Additional file 3. Itemized Cost of Chemoradiotherapy. [file 40463_2021_501_MOESM3_ESM.docx]

| **Variable** | **Mean Cost**  **(CAD)** | **Cumulative Cost n = 2**  **(CAD)** |
| --- | --- | --- |
| Nursing Fee over 3 cycles | $924.00 | $1,848.00 |
| Cisplatin Cost over 3 cycles | $290.70 | $581.40 |
| Medical Oncologist Fee over 3 cycles | $2745.00 | $5490.00 |
| Radiation Therapy (66 Gray in 33 Fractions) | $48,401.00 | $96,802.00 |
|  |  | Total - $104,721.40 |
