# Supplementary figures and images for: Specimen oriented intraoperative margin assessment in oral cavity and oropharyngeal squamous cell carcinoma
Source: J Otolaryngol Head Neck Surg. 2021 Jun 21;50:37. doi: 10.1186/s40463-021-00501-5 (PMC8218466; doi:10.1186/s40463-021-00501-5)

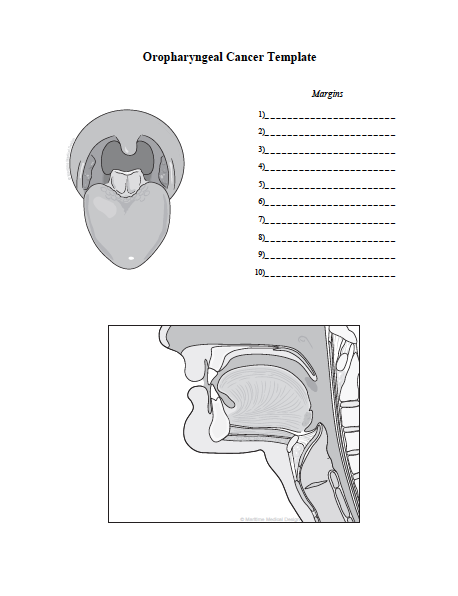

Supplement: Supplementary file 4 — Additional file 4. Oropharyngeal Specimen Orientation Template. [file 40463_2021_501_MOESM4_ESM.docx]
